# Altered connectivity within the salience network during direct eye gaze in PTSD

**DOI:** 10.1186/2051-6673-1-17

**Published:** 2014-11-25

**Authors:** Janine Thome, Paul Frewen, Judith K Daniels, Maria Densmore, Ruth A Lanius

**Affiliations:** Department of Psychiatry, Western University, 339 Windermere Rd, PO Box 5339, London, ON N6A 5A5 Canada; Psychology, Western University, London, ON N6A 5A5 Canada; Department of Psychosomatic and Psychotherapeutic Medicine, Central Institute of Mental Health Mannheim, University of Heidelberg, Medical Faculty Mannheim, Heidelberg, Germany; Department of Psychosomatic Medicine and Psychotherapy, Otto-von-Guericke-University Magdeburg, Magdeburg, Germany

**Keywords:** Posttraumatic stress disorder, Traumatic stress, Childhood abuse, Salience network, Direct gaze, Insula, Amygdala, fMRI functional connectivity analysis, ICA

## Abstract

**Background:**

Posttraumatic stress (PTSD) disorder has been associated with heightened threat sensitivity. Evidence suggests that direct eye gaze leads to sustained activation of the superior colliculus/periaqueductal grey within individuals with PTSD. The present analysis investigated functional connectivity within the salience network (SN) in the same sample as presented in a prior publication during direct versus averted gaze in adults with PTSD related to childhood maltreatment as compared to healthy individuals.

**Methods:**

Functional connectivity within the SN was examined using functional magnetic resonance imaging while participants viewed avatars positioned in direct versus averted gaze relative to the participant in 16 individuals with PTSD related to childhood maltreatment and 16 healthy control subjects. Connectivity within the SN was assessed via Independent Component Analysis (ICA). Associations with symptom severity were explored with multiple regression analyses on individual subject components.

**Results:**

Temporal multiple regression analyses revealed higher connectivity within the SN during direct versus averted gaze which was more pronounced in individuals with PTSD as compared to healthy controls. Compared to controls, individuals with PTSD showed increased integration of the left amygdala and the right insula within the SN. PTSD symptom severity was positively associated with connectivity of the right mid-cingulate cortex within the SN in PTSD subjects only.

**Conclusions:**

Participants with PTSD showed enhanced coupling of the amygdala and the insula within the SN as compared to healthy control subjects during gaze processing. Our results provide evidence for an increased sensitivity of the salience network to direct versus averted gaze in individuals with PTSD related to childhood maltreatment.

## Background

The investigation of social cognition is particularly pertinent because PTSD is often associated with significant interpersonal problems, including interpersonal violence and difficulties forming and maintaining intimate and non-intimate relationship [1–6]. Regulating emotional arising states in a flexible and socially tolerable way are necessary for a successful social interaction. Increasing evidence points to insufficient emotion regulation skills in PTSD subjects [7, 8]. It is important to note that heightened states of fear and anxiety in addition to high levels of social emotions, including guilt and shame weigh heavily on the everyday life of these patients [for review see [1]. However, little is known about the underlying neural mechanism of the latter, e.g. disturbances within early information processing versus higher order information processing.

The majority of behavioural studies investigating social cognition in PTSD focused on deficits in empathic responding, emotion recognition, and theory of mind [9–14]. The capacity to be aware of and comprehend others’ emotions, behaviours, and intentions, that is, theory of mind, may be an important determinant of emotion recognition and empathic responding [15–17].

Response to mutual eye-contact represents one paradigm for studying social cognitive/theory of mind processes in PTSD. Eye contact enables individuals to adopt the perspective of others [18, 19] as it provides a basis for communication and social interaction. A recent review by Senju and colleagues [20] proposed that eye contact modulates cognition and attention. One theory supporting the latter argument is the “communicative intention detector model” [21] which suggests that direct eye contact aims at communicating with the perceiver, resulting in an opportunity to grasp the intentions and emotions of others [21–27]. Steuwe and colleagues [28] investigated the neural underpinnings of response to male avatars, displayed as expressing neutral, happy, or angry expressions, in either direct versus averted gaze relative to the participant. Direct versus averted gaze lead to greater BOLD response within the dorsomedial prefrontal cortex (DMPFC), left temperoparietal junction (TPJ), and right temporal pole (TP) within healthy controls as compared to individuals with PTSD, independent of the emotional expression of the avatar. By contrast, individuals with PTSD demonstrated increased BOLD response within subcortical structures, specifically, the superior colliculus and periaqueductal gray. Enhanced sensitivity of subcortical regions during eye contact processing may compromise a person’s capacity for higher order social cognition. In fact, this subcortical route was described as an “innate alarm system” [29] that facilitates defensive responses, including exaggerated startle, hypervigilance, cowering, and escape [28]. Over activation of this “innate alarm system” may decrease an individual’s capacity for theory of mind and affiliative interaction.

Consistent with the idea of enhanced alarm signaling, a recently published meta-analysis of neuroimaging studies of PTSD demonstrated increased activation across different task types (symptom-provocation and cognitive-emotional tasks) within regions of the salience network, including the right (mid) dorsal anterior cingulate cortex (dACC), and bilateral amygdalae [30]. The authors suggested that increased response within brain regions comprising the salience network may lead to an allocation of cognitive resources to a broad range of external stimuli, thus leading to symptoms of hypervigilance and aberrant goal directed activity.

Three investigations have examined resting state SN connectivity in PTSD [31–33] (for review see also [34]). Generally speaking, these studies demonstrated increased coupling between key nodes of the SN. Specifically, PTSD patients showed an increased coupling between the anterior insula and peri-insula/superior temporal gyrus, hippocampus and amygdala when compared to a combined control group consisting of combat and healthy control subjects [32]. The same investigators also found an increased connectivity between the amygdala seed and the insula in PTSD subjects as compared to combat controls only [31].

Given that previous research indicated enhanced coupling between key nodes of the salience network during resting state in PTSD patients [31–33] combined with our recent findings of enhanced alarm signaling during direct versus averted gaze in this population [28], which may be further related to enhanced downstream activation of the salience network, we were interested in investigating SN associated activation pattern in response to direct versus averted gaze perception in individuals with PTSD related to prolonged childhood abuse, a sample we previously described in Steuwe and colleagues [28]. As a first step, we were interested in examining salience network activation during gaze processing within this sample in general. We therefore applied independent component analysis (ICA). It is a powerful method to characterize large-scale brain networks. So far, ICA based studies have identified components representing different functionally relevant cortical networks such as the SN, the default mode network (DMN), and the central executive network (CEN). Based on the findings described above, we hypothesized enhanced connectivity within the SN during mutual eye contact in patients with PTSD.

## Methods

### Participants and measures

Thirty-two female participants were included in the present study: 16 patients with a primary diagnosis of PTSD related to childhood maltreatment and 16 healthy controls. Clinical and socio-demographic variables were described previously [28] and are presented again in Table 1. PTSD diagnosis was measured via the Clinician-Administered PTSD Scale by a trained psychologist (CAPS, [35]); the CAPS assesses the frequency and intensity of each of the 17 DSM-IV PTSD symptoms [36]. All PTSD patients scored above the common cut-off of 45 for moderate PTSD on the CAPS [35], scores ranging from 50 to 100. To assess comorbid Axis I diagnosis, the Structured Clinical Interview for DSM IV Axis I Disorders [37] was administered. Exclusion criteria were a history of lifetime bipolar disorder, lifetime psychotic disorder, lifetime neurological disorder, current substance use in remission for less than three months, serious head injury and metallic implantations (to account for imaging requirements). In addition, healthy subjects had to be free of any psychiatric disorder. PTSD participants scored higher than controls on the CTQ [38], a well-validated measure used to assess the extent of exposure to traumatic events during childhood and adolescence. Participants with PTSD did not differ from controls regarding age (t_30_ = −0.70, P = 0.490), but significantly less participants with PTSD were currently employed (*χ*^2^_1, 32_ = 5.93, P = 0.015). Participants were recruited via advertisements posted within the community and local mental health treatment centers; all participants provided informed written consent. Study procedures were approved by the Health Sciences Research Ethics Board of Western University, Canada.

**Table 1:** **Demographic and clinical information**

	Controls	PTSD
Demographics		
Mean (s.d.) age	30.56 (12.61)	35.56 (11.63)
% employed (full or part time)	100	68.75
Clinical Characteristics		
Mean (s.d.) CAPS	-	71.50 (15.63)
*Comorbidities (%)*		
Mean (s.d.) present number		
Mean (s.d.) lifetime	0.13 (0.35)	1.88 (1.36)
Alcohol dependence	-	6.25
MDD	-	56.25
Panic Disorder w/wo A goraphobia	-	25.00
Social Phobia	-	12.25
Specific phobia	-	6.25
Generalized Anxiety Disorder	-	6.25
Somatization Disorder	-	6.25
Undifferentiated Somatoform Disorder	-	31.25
Childhood Trauma History		
Mean (s.d.) on CTQ-PA	5.13 (0.34)	10.75 (5.37)
Mean (s.d.) on CTQ-EA	5.94 (1.61)	17.13 (6.23)
Mean (s.d.) on CTQ-SA	5.00 (0)	15.44 (6.36)
Mean (s.d.) on CTQ-PN	5.56 (1.37)	12.06 (4.02)
Mean (s.d.) on CTQ-EN	7.50 (1.37)	16.50 (5.98)

### Behavioral task description and procedure

The stimuli consisted of three-dimensional dynamic animations of four different avatars who moved from horizontally across a computer screen [39]. The experimental set up was modeled on a paradigm by Schrammel et al. (for a detailed description of stimulus material and development, see [39]). The stimulus material consisted of silent video sequences, each 8.8 s, displaying a male avatar moving in profile to the middle of the screen (2 s), then turning either toward the observer directly or at 30° to the left or right, whilst displaying direct or averted gaze, and then moving to exit the screen (2 s). The virtual character either displayed a happy, angry, or neutral facial expression during the period of direct or averted gaze (4.8 s), whereas while entering and exiting the character expressed only a neutral expression. In the case of equally oriented body posture and gaze, this combination was labelled as congruent. In total, 48 video sequences were displayed with four clips for each combination of within-subject factors [gaze direction (two levels: direct versus avert) × congruency (two levels: congruent versus incongruent) × emotion (three levels: happy versus angry versus neutral)] in random order. The male characters were always presented in front of a grey background with only head and shoulders visible. The hair colour (light or dark) as well as the direction of the character’s entrance (from left or right) were counterbalanced. Participants were instructed to pay attention while watching the scenes.

Overall, video sequences were presented in 48 blocks, separated into two runs of 8 min and 54s each; acquiring 178 3 s whole brain imaged volumes. Each run started with a 30-s resting scan and included 24 blocks consisting of 4.7-s arrow condition (an arrow indicated the gaze direction of the avatar), 8.8-s video clip and 7.5-s fixation cross. Video sequences were displayed via an external projector and viewed via a mirror system.

After the scanning procedure, participants were asked to rate the facial expressions of the male characters. The following questions were asked: “What emotion is the character expressing?” and “How do you feel while watching this character?”. Each stimulus was rated on a one to nine (negative to positive) Likert scale.

### Imaging description

All imaging data were collected using a 3.0 T whole-body MRI scanner (Magnetom Tim Trio, Siemens Medical Solution, Erlangen, Germany) with a manufacturer‘s 32-channel phased array head coil. Scanning parameters and preprocessing procedures were previously described (please see [28]).

Orthogonal scout images were collected and used to prescribe a three-dimensional T1-weighted anatomical image of the whole head with 1 mm isotropic resolution (MP-RAGE, TR/TE/TI = 2300 ms/2.98 ms/900 ms, flip angle = 9°, FOV (X, Y, Z) = 256 mm × 240 mm × 192 mm, acc. Factor = 4, total acq. time 3 min 12 s). The anatomical volume was used to determine the angle of the transverse plane passing though both the anterior and posterior commeasures midsagittally and as the source image for interindividual spatial normalization. A set of 64 contagious, 2 mm-thick imaging planes for blood oxygenation level-dependent (BOLD) fMRI were prescribed parallel to the AC-PC plane and positioned to ensure coverage of the top of the brain.

BOLD fMRI images were acquired with the manufacturer’s standard gradient-echo EPI pulse sequence (single-shot blipped EPI) using an interleaved slice acquisition order and tridimensional prospective acquisition correction. EPI volumes were acquired with 2 mm isotropic resolution and the following parameters: FOV = 192 mm × 192 mm × 94 × 94 matrix, TR/TE = 3000 ms/20 ms, flip angle = 90°, 64 slices, 178 measurements.

### Image analyses

Imaging data were preprocessed using Statistical Parametric Mapping 2 (Wellcome Trust Centre for Neuroimaging, London, UK) implemented in MATLAB 7.2 (Mathworks Inc., Sherbon, MA).

To reduce the effects of head motion, each subject’s images were realigned to the first volume of each series. The realigned functional images were then spatially normalized to a standard echo-planar imaging template supplied by SPM2. All images were then spatially smoothed with an 8 mm FWHM Gaussian smoothing kernel.

### fMRI connectivity analysis

Independent component analysis (ICA) is a powerful method to discover the hidden factors from a set of observed data in such a way that they are maximally independent [40]. Previous work implemented ICA successfully to discover either task- or resting-state-related imaging data, suggesting that it is possible to examine temporal coherent networks (TCN) which are associated with more or less external triggered cognitive load [41]).

Hence, group spatial ICA (group-ICA) was conducted for all 32 subjects using the infomax algorithm [42] within the GIFT software (http://icatb.sourceforge.net/, version v2.0e) implemented in MATLAB R2011a (Mathworks Inc., Sherbon, MA). A detailed review of group ICA fMRI analyses can be reviewed in Calhoun et al. 2008, 2009 [40, 41] (see also [43, 44]).

We ran group-ICA on the pooled dataset (all 356 acquired data volumes, concatenated across two runs). In this study, the optimal number of independent components was found to be 20 using modified minimum description length criteria [44, 45]. We applied the ICASSO algorithm implemented in GIFT to increase the robustness of our independent components to initial algorithm conditions by repeating the ICA estimation 20 times. Single subjects’ spatial maps and corresponding time courses were then computed and converted to z-scores for display and use in further statistical analyses. Each voxel in the brain has a z-score representing the strength of its contribution to the component‘s time course [41–46].

### Component identification

The component related to the SN was automatically detected using the spatial sorting function implemented in the GIFT software. In detail, this procedure resulted in a set of spatially independent components, which were correlated with a binary SN mask derived from a previous study [47]. The mask mainly included the ACC, mPFC, mid cingulate gyrus, bilateral STG and, bilateral insular cortices and amygdalae. We selected the component with the highest correlation for further analyses.

### Statistical comparison of spatial maps

For all subjects, z-score spatial maps of the component of interest were imported into SPM8 (http://www.fil.ion.ucl.ac.uk/spm/) for two-sample t-tests. We employed region-of-interest (ROI) analyses for FWE-correction based on the hypothesized effects within the salience network. In particular, we focused on brain regions that have been previously associated with the salience network: cingulate gyrus (MNI coordinates: x = 4, y = −6, z = 34; [48]), amygdala (MNI coordinates: x = −24, y = −4, z = −26; [49] and insula (x = 42, y =10, z = −12; [50]) with a sphere from 6 – 10 mm radius. Statistical significance was assessed using small volume correction [51] at a threshold of P < 0.05 (FWE corrected) for those clusters that also passed a whole-brain uncorrected threshold of P < 0.005 and a cluster threshold of k = 10 [52].

### Statistical comparison of time courses

Multiple regression analysis using the temporal sorting function in GIFT was performed on the ICA time course with the general linear model (GLM) design matrix taken from SPM8. The design matrix contained six regressors: 1) happy facial expression direct; 2) happy facial expression avert; 3) neutral facial expression direct; 4) neutral facial expression avert; 5) angry facial expression direct; and 6) angry facial expression avert. Only congruent body posture and gaze direction was modeled based on our prior analysis reported in Steuwe et al. [28]. This approach leads to a set of beta weights for each regressor, subject and component. To draw inference about the degree of task-relatedness, the utility “stats on beta-weights” implemented within the GIFT software was applied.

Here, beta-weights associated with the component as well as regressors of interest were entered into second-level analyses. To test whether there are differences between groups regarding emotional facial expression and gaze direction, we ran a 2 (PTSD vs Controls) × 3 (happy vs angry vs neutral) × 2 (avert vs direct) ANOVA. In case of significant effects, post hoc t-tests corrected for multiple comparisons were applied. Given that our previous findings focused on direct gaze processing [27], we only report post hoc comparisons for direct greater than avert gaze within the results section.

### Multiple regression analyses of spatial maps

We conducted second-level multiple regression analyses in SPM8 on individual z-score spatial maps within the PTSD group only to assess whether the integration of specific brain regions within the SN is related to symptom severity. The same significance thresholds as described above were applied.

## Results

### Perception of facial expression

**Figure 1:**
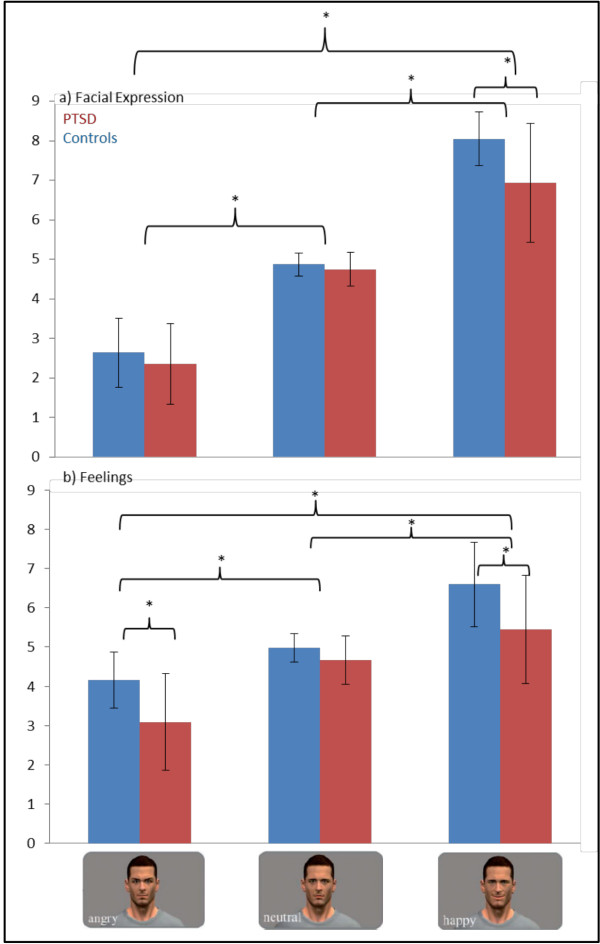
**Means and standard deviations for ratings of the facial expressions and feelings while watching pictures of virtual characters.**

To examine perceived facial expression as well as feelings while observing the virtual characters, two separate 2 (PTSD versus healthy subjects) × 3 (angry versus happy versus neutral) repeated-measures analysis of variance (rm-ANOVAs) were conducted (see Figure 1) and were reported in a prior publication (see [28]). Overall, the analyses of perceived facial expression revealed a significant main effect of group (F_1,30_ = 10.07, P = 0.003) suggesting that PTSD subjects rated happy faces as less happy (t_20.95_ = 2.71, P = 0.013). Across group, a main effect of facial expression was found (F_1.32, 39.69_ = 224.14, P < 0.001) pointing to heightened negative ratings for angry faces as compared to neutral ones (t_31_ = −12.67, P < 0.001) and higher negative ratings for neutral facial expressions than for happy faces (t_31_ = −12.76, P < 0.001).

Analysis of the reported feelings while watching the virtual characters also revealed a significant main effect of group (F_1,30_ = 17.15, P < 0.001) which traced back to the differences in feelings regarding angry (t_24.11_ = 3.01, P < 0.006) and happy facial expressions (t_30_ = 2.63, P < 0.013). In addition, a main effect of facial expression (F_1.35, 40.57_ = 50.83, P < 0.001) indicated that all subjects felt more positive while observing happy expressions when compared with neutral (t_31_ = 5.58, P < 0.001) and angry faces (t_31_ = −7.89, P < 0.001), and participants felt more positive during neutral as compared to angry facial expression (t_31_ = −6.29, P < 0.001).

### Component identification

The spatial sorting revealed a component that closely resembled our SN mask including brain areas previously implicated in the network [32, 48, 49, 53].

**Figure 2:**
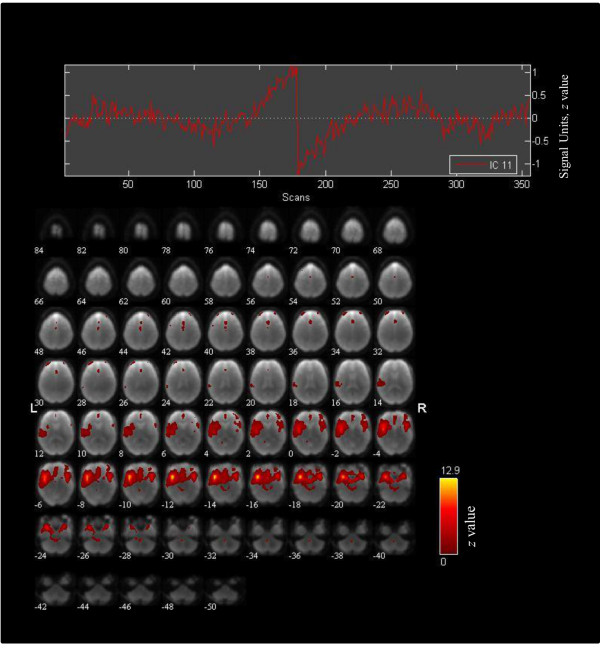
**Independent component analysis component representing the salience network.** The spatial map was identified by GIFT software and correspondent to the mean component estimates of all 32 subjects (PTSD and controls) and resembled the salience network best (r = 0.74).

Component 11 (r = 0.74) (see Figure 2) included mainly the bilateral STG, insular cortices (BA13/14) and amygdalae, but also included smaller clusters within cingulate gyrus and bilateral IFG.

### Statistical comparison of spatial maps

**Figure 3:**
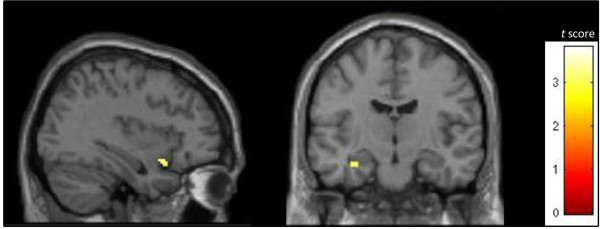
**Between group differences within the salience network.** Compared with healthy control subjects, patients with PTSD showed a higher integration of the anterior insula (p = 0.048, t = 2.99) and amygdala (p = 0.022, t = 3.29) into component 11. Significance is assessed using svc at a tresholded at P < 0.05 (FWE corrected).

Comparisons of spatial maps yielded significant group differences in the integration of the left amygdala (MNI coordinates: −28, −8, −24; t = 3.39) and right anterior insula (MNI coordinates: 38, 12, −16; t = 2.99) into component 11 (Figure 3). Specifically, participants with PTSD showed a higher integration of these brain regions within the SN than healthy controls. We did not identify any brain regions within component 11 that were more integrated within healthy controls as compared to participants with PTSD.

### Symptom severity and spatial distribution

**Figure 4:**
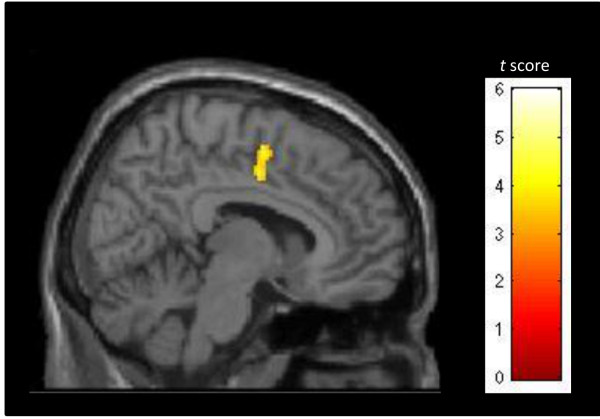
**Correlation between PTSD symptom severity and integration of the mid cingulate gyrus within the salience network.**

To assess whether symptom severity might be associated with specific brain regions within the salience network, second-level multiple regression analyses using PTSD subjects’ z-score spatial maps were conducted. There was a significant positive correlation between the CAPS total scores and the integration of the right mid cingulate gyrus (MNI coordinates: 10, −4, 40; t = 4.46) into component 11, suggesting that PTSD symptom severity seems to be associated with increased recruitment of the mid cingulate gyrus within the salience network (see Figure 4).

### Statistical comparison of time courses

**Figure 5:**
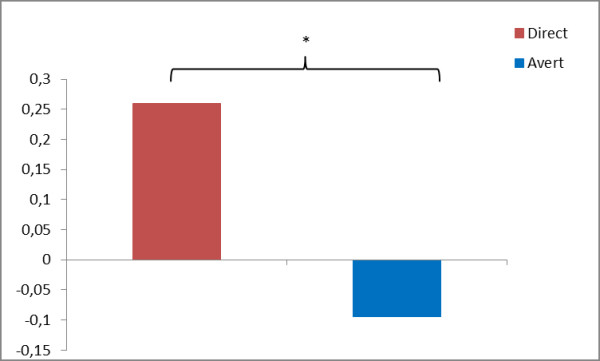
**Effects on gaze direction within the salience network.**

Temporal regression analysis revealed that component 11 showed significant signal change in response to emotion-related gaze direction. The ANOVA of groups and regressors yielded only a significant main effect of regressors (F_5,180_ = 6.11, P < 0.001). Overall, post-hoc t-tests revealed that direct gaze led to a positive signal change within component 11 (t = 3.750, P < 0.001; see Figure 5). More specifically, neutral facial expression led to a higher recruitment of component 11 as compared to happy (t = 3.588, P = 0.0104) and angry (t = 3.157, P = 0.0019) facial expression.

## Discussion

The aim of the present study was to gain insight into SN activity in response to direct versus avert gaze perception in individuals with PTSD related to childhood maltreatment. Overall, analyses revealed an increased coupling within the SN during direct versus averted gaze which was more pronounced in patients with PTSD as compared to healthy control subjects. Specifically, the left amygdala and right insula showed greater integration within the SN in individuals with PTSD.

Moreover, symptom severity as assessed with the CAPS was positively correlated with the coupling of the right mid cingulate cortex within the SN in PTSD subjects.

It has been suggested that the salience network is involved in the detection, identification, and integration of internal and external stimuli which are most relevant for the individual in order to guide behavior that is most suited to maintain homeostasis. It comprises paralimbic structures including the dorsal anterior cingulate cortex (dACC) and orbital frontoinsula [50], brain regions known to be involved in interoceptive-autonomic processing [54–56]. Increased activation in regions associated with the salience network has been associated with the processing of the emotional component of pain [57], empathic responses to pain [58], the experience of metabolic stress, hunger, or pleasurable touch [54], and social rejection [59, 60].

Increased connectivity within the SN during direct gaze may aid the enhanced processing of relevant external cues, i.e., facial expressions, as the activation of this network is associated with heightened attention processing in order to identify relevant cues whether they are cognitive, emotional or homeostatic in nature [50, 54, 61, 62]. In this regard, it is interesting to note that studies demonstrated direct facial expression of anger and happiness to be associated with better performance in emotion recognition tasks as compared to avert facial expression [24, 63–65]. Furthermore, direct gaze when compared to avert gaze seems to increase reaction times for emotion detection [24]. Assuming that increased activation of the SN is triggered by gaze perception, enhanced recruitment of this network may lead to better performance in emotion recognition of facial expressions. The present findings, however, cannot be linked to behavioral performance because participants were instructed to pay attention to the avatars rather than evaluating their emotional expression. Thus, future studies should additionally assess behavioral responses, including the labeling of displayed emotions directly after watching the videos. Interestingly, we found the strongest increase in SN activation in response to neutral direct facial expression. There is evidence that neutral faces are not perceived without an affective component [66], and subjects tend to evaluate such stimuli as negative [67]. Thus, neutral faces may capture more salience processes due to their ambiguous nature.

PTSD subjects appeared to recruit the right insula and left amygdala within the SN more, suggesting increased salience processing during direct gaze perception as compared to healthy control subjects. Healthy participants did not seem to activate structures within the SN more as compared to PTSD subjects as indicated by non-significant spatial results. The latter findings add to the hypothesis that gaze perception triggers the activation of the SN in both groups, although more so in individuals with PTSD related to childhood maltreatment. These results are in line with previous studies showing increased SN coupling during resting-state in subjects with PTSD [31–33] (for review see also [34]). Since the presented sample experienced prolonged exposure to interpersonal violence during childhood, we speculate that the finding of increased connectivity within the SN during direct gaze in trauma survivors may have developed as an adaptive response to interpersonal violence by facilitating enhanced processing of relevant external cues involving facial expression, thus facilitating appropriate survival responses.

Symptom severity as assessed by the CAPS [35] was associated with a higher coupling of the mid cingulate cortex within the SN in PTSD subjects. The mid cingulate cortex is thought to be involved in error detection and attention to behaviorally relevant stimuli [68–72] and has been shown to have functional connections with the insula [50, 73, 74]. Taylor and colleagues [75] examined two systems of connectivity between subdivisions of the insular and cingulate cortices. The authors suggest that one system is involved in emotional salience monitoring and is thought to comprise the anterior insula, posterior anterior cingulate cortex (pACC), and the anterior mid cingulate cortex (aMCC). The second system includes the mid cingulate (MCC) and the entire insular cortex and has been suggested to play a key role in general salience monitoring, including monitoring of the environment, selecting appropriate responses as well as the mapping of skeletomotor body orientation. One may therefore hypothesize a positive association between the latter system and PTSD symptom severity. Future research should examine the functional connectivity between the midcingulate and insular cortex during direct eye gaze in people with PTSD.

Several limitations of the present study are worth noting. Firstly, ICA does not provide information about a direct task related connectivity between unique brain structures but rather enables a characterization of large scale brain networks hidden in a dataset. Here, as a first step, we were interested in an exploration of potential salience network activation within a gaze processing task in general. Future research will need to examine effective connectivity and context sensitive changes in effective connectivity. For this purpose, either psychophysiological interaction analyses [76] which focus on changes in the contribution one brain region has to another while the experimental and/or psychological context is changing or dynamic causal modeling [77] which is also a model-based approach aiming to identify an appropriate neuronal model of interacting brain structures should be employed in future studies. Secondly, only women participated and only male avatars were utilized; future investigations should examine the effects of participant gender in interaction with the gender of the avatars viewed. Based on the present data, we cannot conclude whether the examined salience network connectivity is due to mutual eye contact solely and/or might also be associated with state arousal. Thus, future research should also examine state arousal to disentangle the effects of interpersonal cues and current level of arousal. In addition, eye tracking and pupillary reactivity data during gaze processing should be examined. It will also be beneficial to study a trauma-exposed control group without PTSD in the future; however, we have found it to be very difficult to recruit trauma-exposed subjects who have similar CTQ scores to PTSD groups but who also do not report a history of psychopathology. Finally, the implications of abnormalities in gaze processing for social functioning should be examined in traumatized persons.

## Conclusions

In summary, enhanced coupling of the amygdala and the insula within the SN was observed in individuals with PTSD related to childhood maltreatment during direct versus avert eye contact. These findings suggest increased sensitivity of the salience network during direct eye contact in this population. The behavioral and treatment implications of these findings deserve attention in future research.
